# Utility of Glioblastoma Patient-Derived Orthotopic Xenografts in Drug Discovery and Personalized Therapy

**DOI:** 10.3389/fonc.2018.00023

**Published:** 2018-02-12

**Authors:** Michele Patrizii, Monica Bartucci, Sharon R. Pine, Hatem E. Sabaawy

**Affiliations:** ^1^Graduate Program in Cellular and Molecular Pharmacology, RBHS-Robert Wood Johnson Medical School, Piscataway, NJ, United States; ^2^Rutgers Cancer Institute of New Jersey, Rutgers University, New Brunswick, NJ, United States; ^3^Department of Medicine, RBHS-Robert Wood Johnson Medical School, Rutgers University, New Brunswick, NJ, United States

**Keywords:** glioblastoma, patient-derived xenografts, mouse models, personalized therapy, precision medicine

## Abstract

Despite substantial effort and resources dedicated to drug discovery and development, new anticancer agents often fail in clinical trials. Among many reasons, the lack of reliable predictive preclinical cancer models is a fundamental one. For decades, immortalized cancer cell cultures have been used to lay the groundwork for cancer biology and the quest for therapeutic responses. However, cell lines do not usually recapitulate cancer heterogeneity or reveal therapeutic resistance cues. With the rapidly evolving exploration of cancer “omics,” the scientific community is increasingly investigating whether the employment of short-term patient-derived tumor cell cultures (two- and three-dimensional) and/or patient-derived xenograft models might provide a more representative delineation of the cancer core and its therapeutic response. Patient-derived cancer models allow the integration of genomic with drug sensitivity data on a personalized basis and currently represent the ultimate approach for preclinical drug development and biomarker discovery. The proper use of these patient-derived cancer models might soon influence clinical outcomes and allow the implementation of tailored personalized therapy. When assessing drug efficacy for the treatment of glioblastoma multiforme (GBM), currently, the most reliable models are generated through direct injection of patient-derived cells or more frequently the isolation of glioblastoma cells endowed with stem-like features and orthotopically injecting these cells into the cerebrum of immunodeficient mice. Herein, we present the key strengths, weaknesses, and potential applications of cell- and animal-based models of GBM, highlighting our experience with the glioblastoma stem-like patient cell-derived xenograft model and its utility in drug discovery.

## Introduction

Despite considerable effort that has been dedicated to drug development in oncology, more than 90% of novel therapeutics fail in human trials (1). Undoubtedly, one of the reasons for this high attrition rate is the lack of reliable preclinical models (2). Fast-paced advances in scientific research, technology discovery, and optimization have revealed that cancer is a much more complex disease than previously thought (3). Indeed, the disease is characterized by intense inter- and intra-tumor heterogeneity at the genetic, epigenetic, metabolic, and phenotypic levels. Models are, by definition, in one way or the other imprecise. They are simplified versions of intricate systems and, as such, prone to approximation and partiality. Concordantly, most of the current preclinical cancer models are often unable to recapitulate tumor complexity associated with heterogeneity or predict patient response to treatment (2).

Cell-based assays represent an important pillar of the drug discovery process. Although widely used for testing the therapeutic efficacy of anticancer drugs (4), the clinical value of immortalized cancer cell lines, with some notable exceptions (e.g. U251 GBM cells) (5), as a truly representative tumor model has been continuously questioned (6). Unmanipulated patient-derived tumor cell cultures surely present a more reliable depiction of the tumor features than established cell lines. The 3D culture systems are preferred over the 2D cultures because they mimic tumor conditions *in vivo* (7). Animal models are better representatives of the complex tumor repertoire because they endow a multicellular microenvironment in which patient tumor cells reside, although they are more difficult to establish.

The scientific community has been adapting to the use of patient-derived xenograft (PDX) models, obtained by engrafting patient tumor fragments, or tumor-derived cells into immunocompromised mice. Databases of annotated PDX models, such as the EurOPDX Consortium (8) and the NCI patient-derived models repository (9) have become publicly available. PDXs could maintain the principal histological and genetic characteristics of their originating tumor across early passages (10). Thus, PDXs could be used to improve and direct drug developments toward more personalized approaches. This concept has encouraged the approach of “co-clinical trials,” where PDX models, derived from patients enrolled in a clinical trial, could be used as “avatars” to identify the best therapeutic approach to be administered to patients in real time (11). Still, the use of PDXs in personalized medicine and targeted therapy is challenging, mainly because of the long time required to obtain the preclinical data needed for clinical decision making (10). Nevertheless, the clinical impact of PDX models is not restricted to their use in precision medicine. They can be utilized to gain a better understanding of cancer biology, identify predictive biomarkers for therapy outcome, and could also represent a reliable translational platform for the investigation of the correlation between treatments and genotypes (12) and for identifying novel anticancer therapeutics (13–15). In the next section, we present a concise overview of the currently available preclinical models for glioblastoma multiforme (GBM) and describe our findings on the use of GBM PDX models, which will be invaluable for drug discovery and personalized therapy.

## Preclinical Models for GBM

It is estimated that over 23,000 patients with primary brain cancers were diagnosed in 2017, and over 16,000 brain cancer-related deaths occur annually in the USA (16). GBM, a grade IV astrocytoma, is the most prevalent and lethal primary brain tumor. GBM is classified into four molecular subtypes, depending on the status of PDGFRA, IDH1, EGFR, and NF1 (17). However, all four molecular subtypes were detected in the same patient’s GBM (18), reflecting intense intra-tumor heterogeneity. Only EGFR amplification, IDH1/IDH2 mutations and methylguanine-DNA-methyltransferase (MGMT) hyper-methylation are utilized clinically. GBM patients are typically treated by surgical resection followed initially by radiation therapy plus temozolomide and then with maintenance therapy comprising temozolomide. Despite this therapeutic regimen, 95% of patients suffer from GBM relapse (19). Recurrent GBM patients are treated with the monoclonal antibody Bevacizumab (Avastin), an anti-angiogenic agent targeting VEGF (20). However, the overall survival remains very low, <10% at 5 years, with a median overall survival of merely 14.8 months (21). Because of these dismal survival rates, there is an urgent need for effective therapeutic strategies to prevent or delay GBM recurrence. Thus, it is imperative to have access to reliable preclinical models that can faithfully predict patients’ responses to specific agents.

For the last five decades, investigation of GBM pathogenesis and resistance mechanisms has been carried out using standard cell lines, such as U87, U251, and T98G, among a handful of others, accounting for over 5,000 citations. Yet, these cell models have several limiting imperfections, restricting their reliability in drug development, such as genome and transcriptome adaptation to rapid growth on plastic, as well as alterations and clonal selection caused by culture conditions over many years (6). When injected into rodent brains, these cell lines, except for U251 (5), generally fail to generate tumors with morphological features typical of GBM, such as infiltration into the surrounding brain parenchyma and necrosis. Additionally, a recent genetic analysis of U87 cells (most used GBM cell line) revealed that the DNA profile of the current cell line differs from that of the original cell line established in 1968 (22). For these reasons, the reliability of these cell models is constantly questioned.

## Glioblastoma Stem-Like Cells

There is now a wide consensus among the scientific community about the hypothesis that a dynamic subpopulation of cells within each tumor is responsible for progression and recurrence (23). These cells, endowed with stem-like features, are called cancer stem cells (CSCs) or GBM-initiating cells (GICs). GICs possess unique dynamic features, such as tumor-initiating ability (24), sustained self-renewal and/or intensified clonogenic ability. Abundant evidence corroborates the notion that high grade tumors, such as GBM, are driven by such cells (25, 26), that are therapy-resistant (27, 28) and cause GBM relapse (29). GICs can be isolated from primary tumors based on the expression of specific surface markers, such as CD15 (also called SSEA-1) (30), CD24 (31), and CD133 (32). However, ongoing debates are still challenging the specificity of CD133 as a single marker (33, 34). GICs can also be isolated from tumors using functional assays and grown as neurospheres, using conditions similar to those employed for normal neural stem cell culture (35). The sphere assay (36) is one of such functional assays used to establish GICs expressing stemness factors such as CD133, BMI1, NESTIN, SOX2, SOX9, OLIG2, and ZEB1 (37, 38). When basic fibroblast growth factor and epidermal growth factor (EGF) in sphere culture are replaced with differentiation media, cells undergo differentiation, with loss of stemness factors, and acquisition of mature cell markers (39, 40).

**Figure 1:**
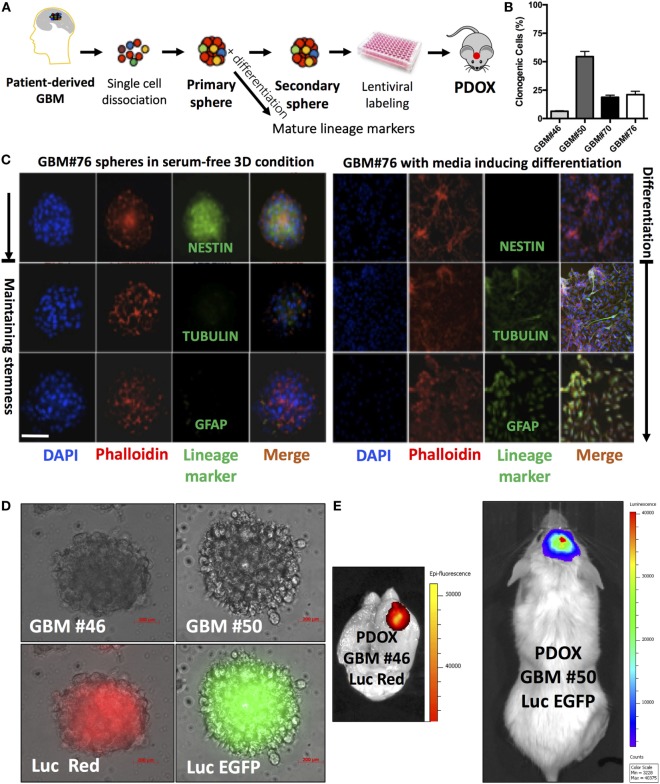
Analysis of neurospheres derived from primary glioblastoma surgical tissues. **(A)** Model of patient-derived sphere assay. Neurospheres are kept in serum-free growth factor condition. To examine their differentiation potential, they are cultured in polyornithine-coated plates. **(B)** Neurosphere clonogenic potential of four different patient-derived glioblastoma multiforme (GBM) cells. **(C)** Immunofluorescence for NESTIN, which is used as a marker of stem cells, being expressed in central nervous system (CNS) stem cells but not in mature CNS cells, and TUBULIN and glial fibrillary acidic protein (GFAP; differentiation markers). TUBULIN-beta-III is a neuron-specific early marker of signal commitment in primitive neurepithelium. GFAP is used as a marker of CNS mature astrocytes and ependymal cells. DAPI is used to label the nuclei, while phalloidin is used to mark the cell architecture within the spheres. **(D)** GBM spheres transduced with lentiviruses expressing either luciferase (Luc) and EGFP or DsRed. **(E)** The two types of transduced spheres were used to generate patient-derived orthotopic xenografts (PDOXs) when injected into NSG mouse brains with 2 × 10^5^ labeled cells and monitored with the IVIS system. Luc was used to longitudinally track tumor growth *in vivo*. Images were from PDOX mice imaged at 8-weeks postinjection. Please note that the image showing Luc and DsRed is zoomed in to demonstrate the brain regions. Color scale was generated with the IVIS software and represents pixel intensities in luminescent or fluorescent images. Color scale units are photons/cm^2^/second. Scale bars are 200 µM in **(D)**.

We successfully generated GIC neurospheres from surgical specimens of consented glioma patients (40) (Figure 1A) through Rutgers Cancer Institute of New Jersey Biospecimen Repository Service using protocols approved by the Institutional Review Board of Rutgers University. By exposing patient-derived dissociated cells to serum-free media supplemented with EGF, FGF, and B27, we generated GIC neurosphere lines from GBM patient samples. Consistent with other studies, these neurospheres are characterized by heterogeneous clonogenic ability (Figure 1B), an aggressive tumor-formation ability and resistance to both radiation and targeted therapy (41). As expected, they possess stem-like properties and can differentiate upon exposure to differentiating media (Figure 1C). This long-term culture method allows the selection of the stem-like population using their functional characteristics (ability to grow in suspension) and responsiveness to niche- (or media)-derived clues (by maintaining stem-like and/or multilineage differentiation abilities) (Figure 1C), rather than relying on sorting cells for the expression of surface markers, whose expression would dynamically change based on the environment and association with stemness features (42). Moreover, in contrast to most standard cell lines, GIC neurospheres could generate tumors when labeled and injected orthotopically into the mouse brain cerebrum (Figures 1D,E), using a protocol approved by the Institutional Animal Care and Use Committee of Rutgers University. These tumor grafts retain the morphological and molecular features of the GBM tumor from which they originated (43). Additionally, the GIC neurosphere model has a recognized clinical relevance and could be used as an independent predictor of clinical outcome in GBM (36). For these reasons, culture of GICs as GBM neurospheres is considered a more representative and reliable cell model compared to standard cell lines. The quest for these models has been exponential, and to satisfy this need, a biobank of GICs, the human glioblastoma cell culture resource, was recently created and made public (44).

## *In Vivo* Models of Glioblastoma

Although cell-based models are of paramount importance for the investigation of GBM pathogenesis, at present, they have limited utility in the context of drug discovery. We cannot fully assess the efficacy of therapeutic compounds without testing them in physiologically relevant systems, such as laboratory animals. *In vivo* models are required especially for diseases affecting the central nervous system, including GBM. In fact, many promising drug candidates for GBM fail during the clinical phases of development due to the blood–brain barrier, limiting or preventing drug permeation to the brain (45). Mice, and to a lesser extent rats, have been the selected species for biomedical research and drug development for gliomas.

There are three main *in vivo* models for primary brain tumors: (1) chemically induced, (2) genetically engineered (GEM), and (3) xenograft (46). Chemically induced models are now considered outdated, since they generate tumors that differ from human gliomas, both at the morphological and molecular levels. On the other hand, GEM and xenograft models have been highly valuable to study the mechanistic determinants of GBM formation and progression and investigate potential therapeutics in the brain tumor field. GEM models are generated based on specific genetic alterations observed in human tumors. In parallel to the increasing understanding of the genetic, epigenetic, and metabolic perturbations involved in GBM and the intensifying advances in genome editing technologies, GEM models have been growing increasingly more complex. Mouse models characterized by conditional and tissue/cell-specific expression of multiple genes simultaneously or developed by altering key signaling pathways involved in GBM (such as Egfr, Nf1, Ras, Pten) are recently used, improving the genetic and phenotypic heterogeneity of mouse gliomas, that are typical of human GBM. *In vivo*, GEM models were proven beneficial to study GICs (47–50), particularly in the clinically relevant immune-competent setting (51).

Lineage-tracing studies provided genetic evidence for CSC-driven clonal growth in several cancers including GBM (52–54). In hGFAP-Cre;Nf1^±^;p53^fl/fl^;Pten^fl/+^ glioma GEM model (100% penetrance in 20–40 weeks), temozolomide could diminish the proliferating GBM cells, while repopulation of GBM was driven by residual quiescent GICs, recruited (to expand and/or divide) after temozolomide was discontinued (53). We reported that Bmi1^+^ cells are highly enriched for GICs (40). Because of their nature, GEM models are an excellent tool to evaluate the role of single genes (or combination of few genes) in GBM onset and progression and could function as ideal models to assess the selectivity of a drug. We are currently using GEM models (47, 49, 53) in lineage tracing studies, by labeling Bmi1^+^ cells (GICs), to study Bmi1 contribution to GICs, cell cycling parameters and small molecule-mediated Bmi1 ablation. GBM resistance to alkylating agents could be driven by deregulation of mismatch and base excision DNA repair pathways (29), which could result from elevated poly-ADP-ribose polymerase (PARP) activation within GICs (55), without altering the proliferative hierarchy of GBM (56), providing a therapeutic opportunity. Resistance to therapy has also been shown to be driven by factors within the microenvironment (57). Moreover, single cell GBM RNA sequencing revealed that GBM phenotypes are influenced by immune cell contributions (18).

The xenograft model is generated by engrafting human cells into immunocompromised animals. The origin of human cells implanted and site of implantation are important considerations, because these conditions determine the formation of different tumors in terms of phenotypical and genetic similarity to human GBM. Since the orthotopic injection site provides the murine stromal support and an environment most similar to the site of the human tumor, it is generally preferred over the heterotopic sites (58). Preclinical studies in the past few decades have been dominated by models created by injecting cell lines subcutaneously into mice. These cells generate xenografts that are mostly dissimilar from the original tumor, both phenotypically and genetically. For instance, genetic analyses of commonly used GBM cell lines revealed how these cells possess DNA profiles divergent from those of human GBM (59). Not surprisingly, standard cell line-derived xenografts, although highly proliferative, lack key features of human GBM such as necrosis and infiltrative nature (5). Thus, due to the clonal selection and genetic drift consequences of adaptation to monolayer culture, cell line-derived xenografts do not satisfy the criteria for a reliable preclinical model. A more representative model of human GBM is the patient-derived orthotopic xenograft models (PDOX), in which dissociated tumor cells or tumor tissue fragments are implanted into mouse brains. As shown recently (60, 61), it is assumed that since these cells or fragments are not cultured *in vitro*, the flaws associated with culture conditions do not technically apply. Another strategy was used to maintain GBM PDXs by serial subcutaneous passaging in nude mice then PDX cells were used generate a panel of GBM PDOXs that, like clinical samples, maintained MGMT promoter methylation (62) and could be used to evaluate temozolomide sensitizing combination therapy (63). Thus, GBM PDOXs largely retain the morphological characteristics of the tumor from which they are derived, such as infiltrative growth patterns, microvascular abnormalities, and necrosis. Moreover, PDOXs also maintain the histological, genetic, and epigenetic features of the parental tumor (60). On the other hand, it has been most recently revealed that selection pressures could lead to distinct tumor evolution between primary human tumors and their corresponding clones propagated in mouse PDXs, including those of GBM (64). Hence, PDOXs, within early passages and/or with an established genomic parity with their corresponding primary GBM, represent a more superior model compared to GEMs in the sense that PDOXs more faithfully resemble human GBM.

## Analyses of the PDOX Model of Glioblastoma

Despite the advantages of GBM PDOXs, they are far from perfect. First, the tumor-establishment rate of this model is highly variable, ranging from a reported 80–90% success rate (60) to a complete failure in generating GBM PDOXs (65), especially when small tumor fragments are the source material (65). Second, the initial engraftment has a long latency (2–11 months) (66), and could potentially fail to progress, thus limiting their use for the “co-clinical trials” approach. Third, there is high variability between PDOXs in terms of clonal composition of the tumors. This could represent an advantage, in the sense that different PDOXs reflect the inter-tumoral heterogeneity observed in GBM patients. However, such heterogeneity does not favor experimental standardization, thus challenging the use of PDOX models in drug development.

Other alternatives to the “classic” PDOX models have been proposed, realized by adding an *in vitro* step. One such model is represented by injection of spheres (66), the second is by injection of organoids (67), usually derived from resections of GBMs from patients, and both spheres and/or organoids could also be generated upon initial propagation of PDOXs in mice. The biopsy tissue is minced and exposed to matrix-coated culture vessels in serum-free media. Injection of GBM spheres yields a tumor take rate close to 100%, thus significantly improving on the take rate of PDOX models (66). Although these models require validation of genomic, epigenomic, metabolic and expression parity with the original GBM (because tumor specimens are exposed to culture), in our opinion, when generated and paired with defined clonal tumors and/or single cells reflecting the original tumor diversity, they provide a way to refine and further exploit these models. Indeed, these alternative approaches offer the advantages of the use of the 3D spheres and organoids in drug screening and selecting personalized therapy, while still supporting the development of reliable PDOXs.

**Figure 2:**
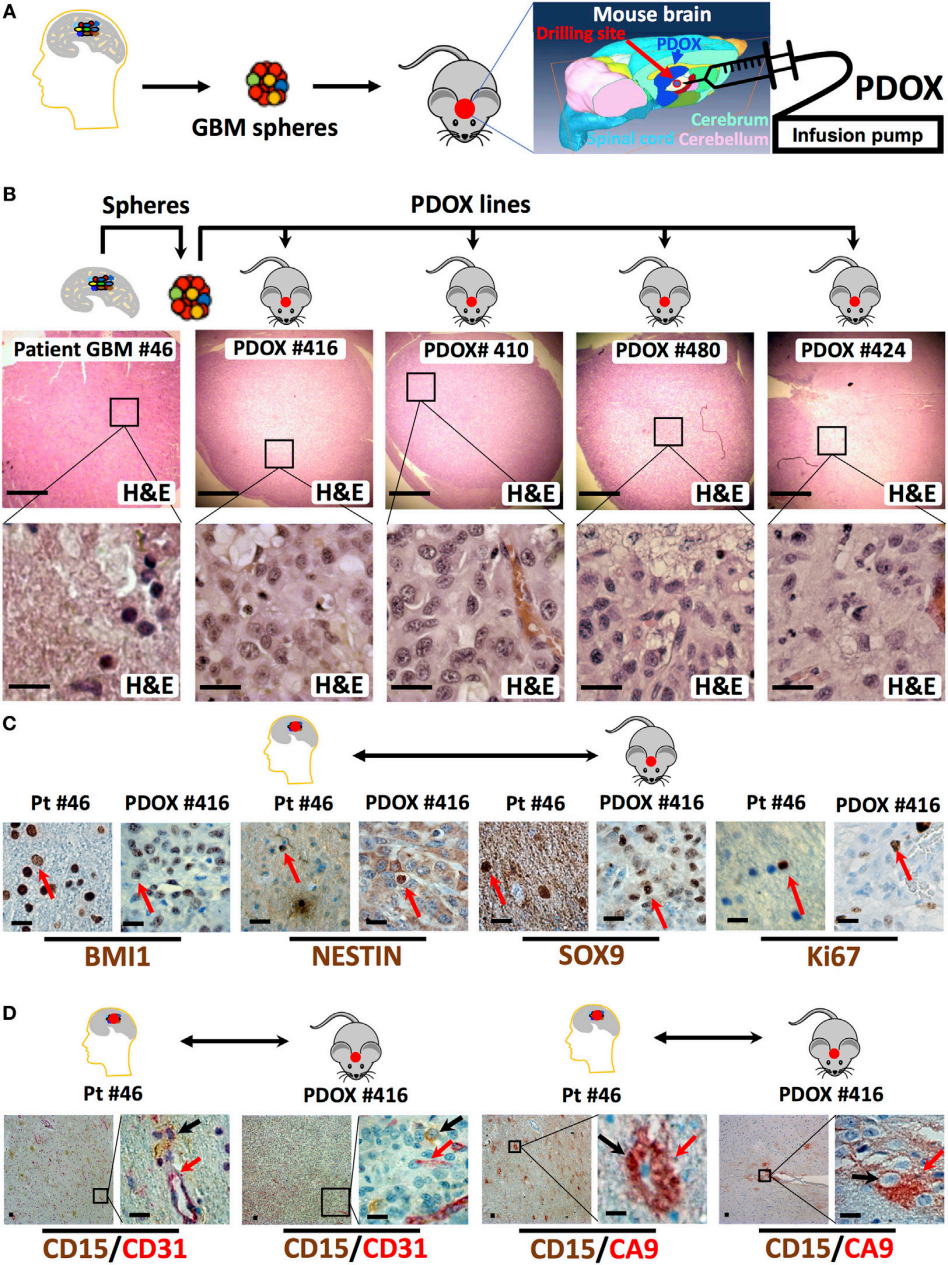
Histologic and immunophenotypic parity between original glioblastoma tissue and patient-derived orthotopic xenografts (PDOXs). **(A)** Strategy to generate spheres and PDOX from primary glioblastoma tissue. The diagram displays the process of microinjecting sphere cells into the cerebrum region of the mouse brain. The location of burr drilling hole (red circle) in NSG mice skull is demonstrated for microinjections using stereotactic infusion pump resulting in effective (90% take) generation of orthotopic glioblastoma multiforme (GBM) PDOXs. **(B)** Histological H&E analysis of original patient-derived glioblastoma tissue (patient (Pt) #46) and four different PDOX lines generated from the same patient-derived spheres. Note that the cell density is different in these sections as it depends on the number of cells engrafted into the PDOXs. The lower panels are 1,000× higher magnification of the outlined areas in the top panels. **(C)** Representative sections for comparison of the expression of stem cell proteins (BMI1, NESTIN, and SOX9) and the proliferation marker Ki67 in the original patient GBM tissue and the corresponding PDOX. Red arrows indicate positive cells. **(D)** Representative sections for comparison of the clustered expression of the GBM stem cell marker (CD15) in vascular niches and with the hypoxia protein carbonic anhydrase IX (CA9) near blood vessels expressing CD31, both in the original patient GBM tissue and the corresponding PDOX. Black and/or red arrows indicate positive cells. Scale bars are 500 µM in the upper panel of **(B)** and 20 µM in the lower or magnified panel of **(B–D)**.

In our experience, the time needed for the initial engraftment of the GIC neurospheres generated in our lab (40) ranged between 2 and 6 weeks. To longitudinally track tumor growth *in vivo* without sacrificing the engrafted animals, we transduced GIC neurospheres with lentiviruses encoding Luciferase and either EGFP or DsRed before slow-release injection with an infusion pump, thus allowing us to precisely control the site of injection at the mouse brain cerebrum and the growth rate through bioluminescence and/or fluorescence *in vivo* monitoring (Figures 1D,E and 2). Just two months after injection of as low as 10^4^ GICs, most animals showed neurological signs associated with tumor burden. PDOXs recapitulated key GBM morphological features (Figure 2B), including necrosis and overall expression of the stem cell proteins BMI1, NESTIN, and SOX9 and the proliferation marker Ki67 (Figure 2C). Moreover, PDOXs demonstrated a high degree of hyperplastic blood vessels, a hallmark of GBM (68) representing the original GBP patient tissues (Figure 2D). Additionally, GBM cells expressing the GIC CD15 (30) were present in clusters of GBM niches in proximity of blood vessels, and cells expressing the hypoxia protein carbonic anhydrase IX were in close proximity to cells expressing CD15, within both the original GBM patient tissue and the corresponding PDOXs (Figure 2D). Our studies demonstrate that PDOXs generated from patient-derived GIC neurospheres could make a reliable model for developing targeted and personalized therapies for GBM patients.

Nevertheless, even this model is not flawless. First, although proliferating spheres can be easily obtained within 1–2 weeks of culture, the success rate for their generation is highly variable, ranging from 10 to 100%, and like PDXs, depending on the laboratory expertise and, most likely, on the nature of the initial human specimen (69). Second, experimental standardization remains challenging, given the variability among xenografts generated from different patients. Third, like other models involving manipulation of the original tumor before injection into the animal, there might be a selection of a specific subpopulation (in this case of the one endowed with stem-like features) that could result in an under-representation of the genetic and functional cellular heterogeneity found in the patient’s tumor. However, we believe that selection of GICs could be a more accurate way to generate an animal model aimed at testing therapeutics to target therapy resistant cells and/or prevent GBM recurrence. The first-line of treatment for GBM patients is the surgical removal of the tumor mass. Since many studies confirmed that GICs are responsible for tumor recurrence (26, 29) and GICs regulate GBM molecular heterogeneity (70), the use of PDOX and/or PDX clinical trials (12), to develop strategies to target GICs may be more effective in overcoming therapy resistance caused by intratumoral heterogeneity (71). The PDOX models generated with genomically defined GICs could be a more ideal model to develop and test such therapeutic strategies tailored for each patient and/or pointed toward prevention or delay of recurrence.

## Conclusion and Future Directions

Here, we presented a concise overview of the available preclinical models of GBM and explained their benefits and pitfalls. Although all described models have their unique utility, we can assert with confidence that some are better than others, especially in the context of testing therapeutics aimed at preventing GBM recurrence. Within this scope, the model of election is represented by PDOX models, which allow, to a certain extent, the recreation of the genetic, histologic, and morphologic profiles of human GBM. Particularly, our experimental work and that of other labs showed that, if preventing or delaying recurrence is the goal, GIC neurosphere-derived models are excellent. However, although informative, this model can still be largely improved by addressing some of its major limitations. For instance, we should consider the cellular plasticity associated with GICs (72), and evaluate how heterogeneity and plasticity influence generation of neurospheres or organoids *in vitro* (73). Moreover, solutions to address the experimental standardization of PDOX would be desirable, especially for testing antineoplastic agents. Mouse PDOX could be preceded with rapid screening assays such as the zebrafish PDX assay using fewer cells and multiple clones from the same tumor (74) to screen for response to single and/or combination therapy tailored against patient’s specific perturbations, as we (14, 75, 76) and others (77) have recently shown, could shorten the time required to obtain the preclinical data needed for clinical decision making in precision medicine. Additionally, GBM PDOX and PDX models of other cancers suffer from the same limitations in terms of under-representation of the human cellular stromal and immunologic contribution to therapy outcome. The use of “humanized” mice (expressing components of the human immune system) and the co-injection of human stromal/immune cells and human cytokines, together with patient-derived cancer cells might solve this later limitation (78).

To maximize the utility of patient-derived GBM models, both the scientific investigation and the drug discovery process should consider the characteristics of the different models and utilize the most appropriate one for the specific scientific question/investigational goal to be addressed. The use of multiple models is likely to be required to comprehensively assess treatment efficacy. If we do not fall into the temptation of generalizing the findings obtained from one model to the whole field of cancer investigations, GBM preclinical models can be very informative, even at their present state.

## Ethics Statement

Glioblastoma studies were performed using surgical specimens of consented glioma patients collected through Rutgers Cancer Institute of New Jersey Biospecimen Repository Service using protocols approved by the Institutional Review Board of Rutgers University. Animal studies were conducted using a protocol approved by the Institutional Animal Care and Use Committee of Rutgers University.

## Author Contributions

MP: designed research, performed research, analyzed data, and wrote the manuscript. MB: designed research, performed research, analyzed data, and edited the manuscript. SP: designed research, analyzed data, and edited the manuscript. HS: designed research, performed research, analyzed data, wrote the manuscript, and supervised the study.

## Conflict of Interest Statement

The authors declare that the research was conducted in the absence of any commercial or financial relationships that could be construed as a potential conflict of interest. The reviewer SL and the handling editor declared their shared affiliation.
